# Structural Features of Small Molecule Antioxidants and Strategic Modifications to Improve Potential Bioactivity

**DOI:** 10.3390/molecules28031057

**Published:** 2023-01-20

**Authors:** Nathan C. Charlton, Maxim Mastyugin, Béla Török, Marianna Török

**Affiliations:** Department of Chemistry, University of Massachusetts Boston, 100 Morrissey Blvd, Boston, MA 02125, USA

**Keywords:** ROS/RNS, antioxidant, polyphenols, anilines, hydrazones, Schiff-bases, thiols, isoprenoids, strategic modifications

## Abstract

This review surveys the major structural features in various groups of small molecules that are considered to be antioxidants, including natural and synthetic compounds alike. Recent advances in the strategic modification of known small molecule antioxidants are also described. The highlight is placed on changing major physicochemical parameters, including log *p*, bond dissociation energy, ionization potential, and others which result in improved antioxidant activity.

## 1. Introduction

### 1.1. Role of Antioxidants in Redox Homeostasis

Free radicals are an integral part of normal physiological functions such as cell signaling, immune response, and important contributors to maintaining general redox homeostasis [1,2,3]. Reactive Oxygen and Nitrogen Species (ROS/RNS) are natural products of cellular metabolism. They are beneficial against infectious agents and participate in signaling pathways as so-called “redox messengers”; however, they can induce harmful oxidative stress as well [4]. The radical activity is controlled by the combination of endogenous and exogenous antioxidant systems [5,6,7,8]. When these systems malfunction, the excess radicals result in extensive oxidation and damage to a range of biomolecules that can lead to dysfunction or cellular death [9]. Free radical damage appears to be common in many neurological diseases, such as Alzheimer’s disease, Parkinson’s disease, and multiple sclerosis [10,11,12,13,14]. It also contributes to aging, cancer, diabetes, high blood pressure disorders, and cardiovascular diseases [11,15,16,17]. Many exogenous small molecule antioxidants such as polyphenols [18,19] are commonly isolated from a broad variety of plants [20], and their potency is extensively studied [21,22,23,24,25,26,27]; however, their bioavailability is frequently poor [28,29]. In addition to polyphenols, carotenoids, amines, betains, and betalains are also potent naturally occurring small molecule antioxidants [30,31], although much less frequently investigated [32,33]. Advances in the isolation, characterization, and bioactivity of antioxidants have been subjects of recent reviews [7,34,35,36,37,38,39]. Here, we define small molecule antioxidants as structurally well-characterized individual compounds regardless of molecular weight. Thus, polymeric biomolecules, such as enzymes, will not be reviewed.

### 1.2. Application of Antioxidants as Therapeutics and Food Supplements

Due to the health benefits of antioxidants, food products rich in antioxidants are often called “superfoods” [40,41]. The so-called “French Paradox” especially increased interest in dietary small molecule antioxidants after it was noted that despite high saturated fat and alcohol intake, Mediterranean populations are less likely to develop coronary heart disease [42]. This has been attributed to their relatively high consumption of red wine, which is known to be rich in antioxidants, particularly flavonoids. Although evidence is limited, this has not stopped extensive support for the idea that red wine and certain superfoods will lower risk of cardiovascular disorders or cancer [43,44,45]. In addition, pharmaceutical therapies using antioxidants to treat various diseases have also been proposed or established [46,47,48]. These therapies act via different mechanisms. In this work we will focus on direct ROS/RNS scavenging.

### 1.3. Major Mechanisms of Radical Scavenging

Direct scavenging is the most studied mechanism, and many of the well-known dietary antioxidants discussed here rely on this mechanism. Typical ROS/RNS quenched include hydroxyl radicals, peroxyl radicals, hydrogen peroxide, peroxynitrite radical, and singlet oxygen, among others [36].

Direct radical scavenging works through two mechanisms in particular: Single Electron Transfer (SET or simply ET) and Hydrogen Atom Transfer (HAT). In the HAT mechanism (Equation (1)), the antioxidant reacts with the free radical transferring one of its hydrogen atoms to the radical via homolytic rupture of an -OH bond.

(1) $$ArOH+R^{∙}\to {ArO}^{∙}+RH$$

In the SET mechanism (Equation (2)), a single electron is transferred from the antioxidant to the radical, ionizing both compounds in the process.

(2) $$ArOH+R^{∙}\to {ArOH}^{∙+}+R^{-}$$

Although HAT can form a new radical, and SET ionizes both the antioxidant and the former radical, the new species are far less reactive than the original radical, due to resonance stabilization [49]. Some antioxidants bind directly to the radicals, forming radical-substrate complexes.

There are also mixed mechanisms where the boundaries between HAT and SET can be difficult to identify. The solvent can affect which mechanism is favored; protic solvents appear to impede HAT reactions in some assays [50,51].

In addition, there is the two-step sequential proton loss electron transfer (SPLET) mechanism (Equations (3) and (4)), which has been shown to play a role in flavonoid ionization and can only occur in ionizing solvents [52].

(3) $$ArOH\to {ArO}^{-}+H^{+}$$

(4) $${ArO}^{-}+{ROO}^{∙}\to {ArO}^{∙}+{ROO}^{-}$$

### 1.4. Antioxidant Assays

**Figure 1:**
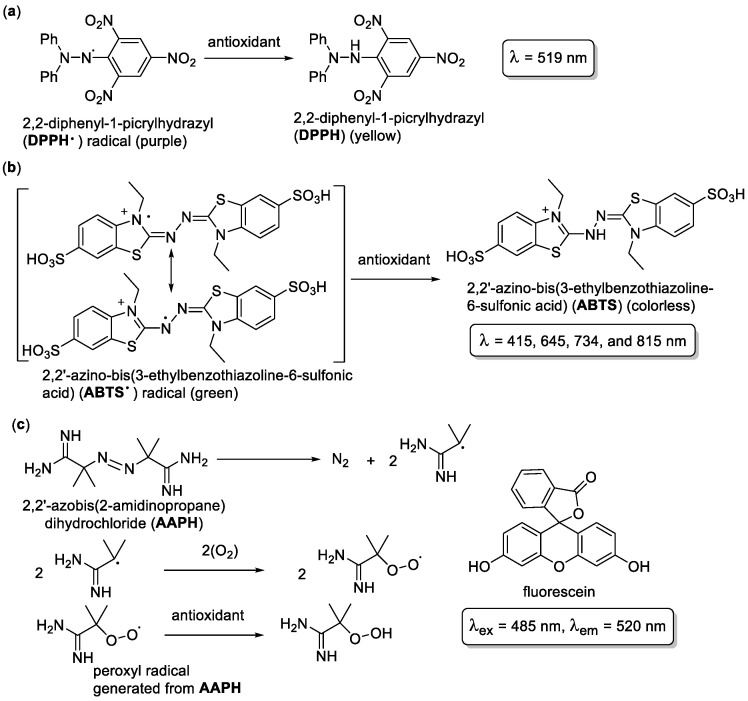
Chemical reactions occurring during common assays to characterize the activity of radical scavengers involving stable and in situ prepared radicals. (**a**) DPPH assay; (**b**) ABTS assay and (**c**) ORAC assay.

There are several antioxidant assays that characterize the potency of radical scavengers, such as the DPPH (2,2-diphenyl-1-picrylhydrazyl), the ABTS (2,2′-azino-bis(3-ethylbenzothiazoline-6-sulfonic acid), and the ORAC (Oxygen Radical Antioxidant Capacity) assays, the total radical trapping antioxidant parameter (TRAP) method, the crocin bleaching assay (CBA), the total phenols assay by Folin-Ciocalteu reagent (FCR), the ferric ion reducing antioxidant power (FRAP) assay, and a total antioxidant capacity assay using a copper(II) complex (CUPRAC) [53]. While all of these are broadly used, the majority of the results compiled in this work are based on the three most commonly used assays: DPPH, ABTS, and ORAC [36,54]. These assays work in a similar fashion: the tested compound is exposed to a solution of a specific radical species, which either contains a dye or a colored radical. If a colored radical is used, reducing the radical will result in color loss, which can be measured via UV-Vis spectrophotometry. If a fluorescent dye is used, the fluorescence of the solution over time will be measured. The results are often listed as IC_50_/EC_50_ (concentration of the scavenger that reduces 50% of the radicals), or trolox equivalent (the concentration of a common reference antioxidant trolox (6-hydroxy-2,5,7,8-tetramethylchroman-2-carboxylic acid) required to reduce the radicals to the same extent) [55], although other equivalent reference compounds such as ascorbic acid or resveratrol are also used in assays. It is also not uncommon to see the percent of the radical scavenged or even the percent of inhibition given as a way of comparing the activities of various compounds. The chemical schemes of the assays are depicted in Figure 1.

The DPPH assay (Figure 1a) is a HAT-based assay measuring the scavenging activity of antioxidants against the commercially available DPPH radical. Since the radical is purple and the reduced form is yellow, a decrease in the absorbance at 519 nm characterizes the activity of the compound compared to the control DPPH solution (Figure 1a). The reaction times are compound dependent in the few minutes to several hours range [56]. The ABTS/TEAC (trolox equivalent antioxidant capacity) assay is used for both HAT and SET mechanism-based antioxidants. As the ABTS radical is reduced, it undergoes a color change from an intense green to colorless, allowing for a simple colorimetric analysis (Figure 1b) at 415, 645, 734, and 815 nm. The ABTS radical is generated in situ, and typical reaction times are under 30 min. The results are given in trolox equivalents, with a larger number being more favorable (e.g., an antioxidant with a TEAC of 2.3 means that 1 mmol of the compound is as effective as 2.3 mmol of trolox) [56]. The ORAC assay (Figure 1c) is a HAT-based assay that uses the 2-hydroperoxy-2-methylpropanimidamide radical, which is much smaller than the DPPH and ABTS radicals. This assay will measure the antioxidant’s ability to scavenge peroxyl radicals generated by AAPH in situ. Fluorescein dye is used to determine the extent of scavenging. The dye is oxidized by the peroxyl radical; the presence of the antioxidant prevents the fluorescein from oxidation. The activity is measured by exciting the sample at 485 nm and measuring the decrease in fluorescence at 520 nm over time. The ORAC value compares the fluorescence of the tested solution to that of a control sample, using a trolox-tested sample and is typically reported as a trolox equivalent [56]. Ascorbic acid is also often used as a reference compound to compare the radical scavenging abilities of various antioxidants using the vitamin C equivalent antioxidant capacity (VCEAC) assay [57,58].

## 2. Pharmacophore Features and Substituents Important to Antioxidant Activity

There are four main groups of structural motifs that contribute to the antioxidant activity of small molecules. These include highly conjugated (e.g., aromatic) hydroxyl groups, amino groups, thiol groups, and isoprenoid groups. Many compounds contain more than one of these features. In this work, we describe each group individually and give representative examples of structures and activities for each of them.

### 2.1. OH Group

Highly conjugated OH groups are the most common pharmacophore features in antioxidants. These antioxidants operate by the HAT and SET mechanisms and in combination as well, with specific conditions often favoring one mechanism over the other. The structures of OH-based antioxidants vary greatly, ranging from simple molecules like ascorbic acid [59] to polyphenols [60], or compounds with long alkyl chains such as in vitamin E. The most common structure, however, is aromatic in nature, and most known OH-based antioxidants are phenols. Others, such as ascorbic acid, while not aromatic, do offer a high degree of conjugation in order to stabilize radicals [61].

### 2.2. NH Group

Nitrogen-based antioxidants possess a conjugated active NH group as their pharmacophore with mechanisms, similar to that of OH-based compounds [62]. Many of these compounds are aromatic as well, or at least highly conjugated and act via the HAT, SET, and combined mechanisms with the hydrogen in the aromatic amino group acting as the donor. Common examples of NH-based antioxidants are anilines, melatonin, bilirubin, imines, hydrazones, and betalains.

### 2.3. SH Group

Thiol-based antioxidants have an SH group as the reactive unit and include some of the most common endogenous antioxidants, which play critical roles in cellular redox reactions by regulating the composition of disulfide bonds in proteins [63]. These HAT-type antioxidants are linked to the reduction of oxygen in biological systems, the main form of the antioxidant activity of thiols. Reducing ROS to less harmful species thiols undergo oxidation to disulfides (Equation (5)) [64].

(5) $$2R-SH\to R-SS-R+2e^{-}+2H^{+}$$

Thiols are also known to have good metal chelating abilities and have been used for their ability to remove heavy metals from biological systems. Common examples of thiol-based antioxidants are glutathione, penicillamine, and cysteamine [65].

### 2.4. Carotenoids

While carotenoids do not contain specific XH groups, they are effective quenchers of singlet oxygen and peroxyl groups. This activity comes from their highly conjugated isoprenyl chain that allows enhanced resonance stabilization of radicals. They are believed to work via the HAT mechanism. Compounds such as vitamin A and β-carotene are known carotenoid antioxidants [66].

### 2.5. Compound with Combined Features

Several antioxidants work via a combination of the above-described pharmacophore features. Examples of these include MitoQ or Coenzyme Q10 which contain long lipophilic tails with an active XH group [67,68].

## 3. Representative Examples of Major Antioxidant Categories

### 3.1. OH-Containing Antioxidants

Hydroxyl groups in a conjugated environment are known to provide antioxidant activity; for example, polyphenols rely on them for their activity. It is one of the most important and common pharmacophores [69,70]. Studies clearly illustrate this; compounds lacking such hydroxyl groups (e.g., flavone) have negligible activity while polyphenols exhibit significant activity [71]. Increasing or decreasing the number of hydroxyls in a molecule affects the overall radical scavenging activity accordingly. Functionalization (e.g., methylation) of the hydroxyl groups also results in a steep drop in the antioxidant activity, demonstrating that the mobile H is essential for it [72].

#### 3.1.1. Ascorbic Acid

**Figure 2:**
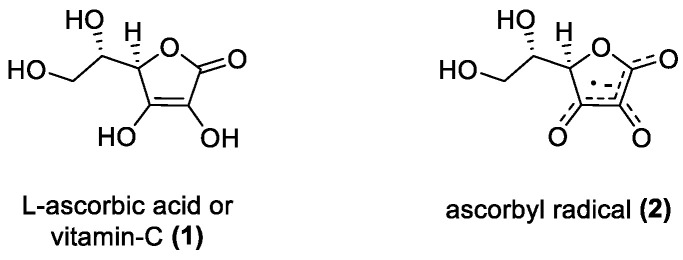
Structure of ascorbic acid and the ascorbyl radical depicting resonance stabilization.

Ascorbic acid (**1**) or vitamin C, is a water-soluble antioxidant and a cofactor of multiple enzymes [59]. It is a six-carbon lactone, an enol, and effective radical scavenger. A relatively stable non-reactive radical that forms upon radical scavenging action makes it an ideal antioxidant (Figure 2) [73]. Although it is nonphenolic, the dienol and lactone ring conjugation allows it to form a stable radical thus preventing it from being reduced or oxidized generating more radicals, making it ideal for breaking radical propagation [74,75]. One important mechanistic aspect of its antioxidant activity is that it can donate electrons to a wide range of other compounds and thus regenerate the antioxidants in the lipophilic vitamin E family, which can prevent Low Density Lipoproteins (LDL) peroxidation [76,77]. Ascorbic acid can interact with α-tocopherol at a membrane surface and converting the tocopheroxyl radical to its hydroxyl form, essentially recycling it, and the more stable ascorbyl radical (Figure 2) is enzymatically reduced [75], preventing lipid oxidation much more effectively, than in systems without ascorbic acid [78]. However, these ex vivo observations were not yet confirmed in vivo [79], although a double-blind study found that supplemental vitamin C reduced lipid peroxidation in humans [80]. Other studies have also shown that ascorbic acid works primarily in conjunction with other antioxidants to increase the overall efficiency of those compounds [81,82,83,84]. Due to its structure, the strong electron donating ability shows that its potential lies more in an assistive role in regenerating antioxidants, and since its radical is relatively unreactive, it can help terminate harmful chain reactions.

#### 3.1.2. Phenolic-OH Containing Antioxidants

**Figure 3:**
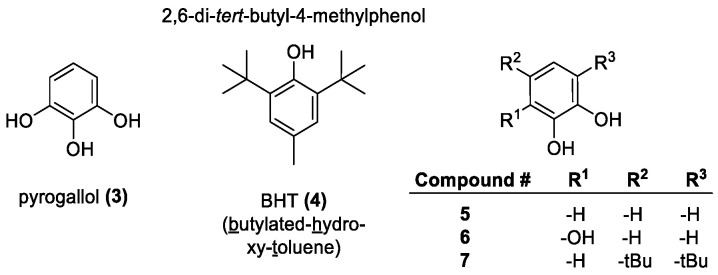
Structure of common phenol-based antioxidants [87].

Phenols, the most common natural antioxidants, are composed of hydroxyl groups attached to aromatic hydrocarbon rings. The aromatic nature of phenol allows for any radical or ion formed on the hydroxyl group to be resonance stabilized. Polyphenols are known to be much more readily oxidized than monophenols. The antioxidant action of phenols occurs by the HAT and SET mechanisms [85]. The antioxidant activity is affected by the number and the arrangement of the hydroxyls on the aromatic ring [86]. Several typical phenol-based antioxidants are shown in Figure 3.

##### Phenolic Acids

**Figure 4:**
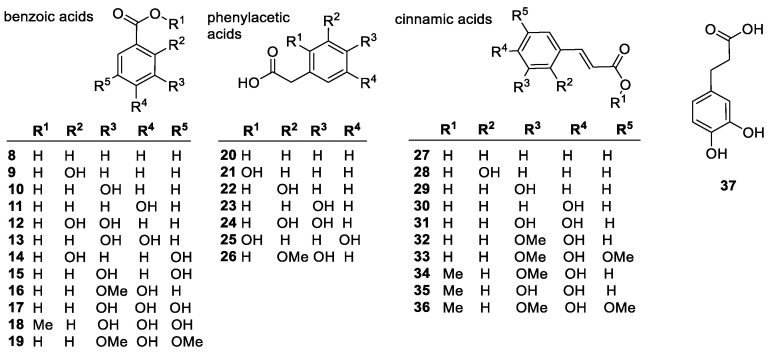
Structure of representative phenolic acid-type compounds with reported antioxidant activity.

Phenolic acids are aromatic carboxylic acids; such as benzoic acids, phenylacetic acids and cinnamic acids. Some common derivatives are shown in Figure 4. According to the reports, *meta*-hydroxyl substitution on monohydroxybenzoic acids has high activity, while the *ortho*- and *para*-isomers are inactive. This is due to the electron-withdrawing (EW) properties of the carboxyl group, the *meta*-position (**10**) is the least sensitive to this deactivation [61]. It was also found, however, that they have good hydroxylation potential and are effective radical scavengers, even in vivo, as shown by studies with the otherwise weak scavenger salicylic acid (*ortho*-hydroxy substitution) [88]. Dihydroxyl benzoic acids have significantly higher antioxidant activity, predictably, the *meta*-arrangement, offering good results. The 3,5-hydroxyl substitution offers the best activity; the double *meta*-substitution acts as a strong counterbalance to the EW effect of the carboxyl group. A *meta*-methoxy group was also found to increase activity in a 3,4-arrangement. The increasing number of hydroxyl groups also has a positive effect on activity; triols having significantly increased antioxidant potential. The gallic acid isomer containing three hydroxyl groups has the highest activity of the common phenolic acids and is also often used to modify other phenolics, as seen with epigallocatechin (vide infra). Adding a carbon or carbon chain between the carboxyl and the phenyl ring is known to increase activity. This is because the further away the electron-withdrawing group is from the ring, the more easily hydrogen can be abstracted.

Phenylacetic acids contain a CH_2_ link between the phenyl ring and the carboxylic acid group. As a result, the carboxylic acid group only influences the antioxidant activity through the mild inductive effect [89]. Since the -CH_2_COOH substituent is a weak electron-donating group, the electron density over the phenyl ring increases somewhat, thus decreasing the bond dissociation energy (BDE) of the phenolic OH bond increasing the antioxidant activity of the compounds [90].

Gallic acid (**17**) is perhaps the most important of the phenolic acids due to its broad range of physiological effects, from antimicrobial, anti-inflammatory, and anti-cancer activity to its ability to prevent oxidative damage. Since it is partially hydrophobic, it shows some protection against lipid peroxidation. Derivatization of some of the hydrophilic groups improves lipophilicity and allows for better overall lipid permeability [91]. In ester derivatives with the same distribution of hydroxyl groups, longer chains were more hydrophobic allowing for greater permeation although showing lower activity. It suggests that steric effects are impeding the mobility of the compounds, and that longer chains cause more steric hindrance, despite being better suited for lipid permeation [92].

Cinnamic acids (**27**–**36**) possess a C=C double bond between the phenyl ring and the carboxylic acid group. The *para*-hydroxy substitution results in the highest activity since the C=C bond shields the ring from the EW effect of the carboxyl group. Common cinnamic acid derivatives include: coumaric (**30**), caffeic (**31**), sinapic (**33**), and ferulic acids (**32**). The growing number of hydroxyl groups will increase activity, as shown with ferulic acid being less active than caffeic, but sinapic acid is the most active due to the two *ortho*-methoxy groups (to the OH). While esterification of the carboxylic acid typically has minimal effect, the methyl ester of sinapic acid (**36**) does show higher activity [93]. Esterifying ferulic acid (**34**) resulted in a decrease in activity in DPPH assays, albeit minimal, except in inhibition of oil oxidation, when a slight activity increase was observed [88,94]. Protection against lipid peroxidation in egg yolk liposomes decreased significantly when the esterifying alkyl chains were larger than 3-carbons. The same effect was found with gallic acid (**17**) as well. Ferulates (derivatives of **32**) worked better with larger and bulkier ester groups, but gallic acid showed a dip in performance with the lauryl vs. stearyl esters, suggesting an optimum chain length before steric effects start hindering performance. In the same assay, caffeic acid was the most active overall, and *p*-coumaric acid had similar results to ferulic acid, but their esters were not tested [94].

##### Coumarins

**Figure 5:**
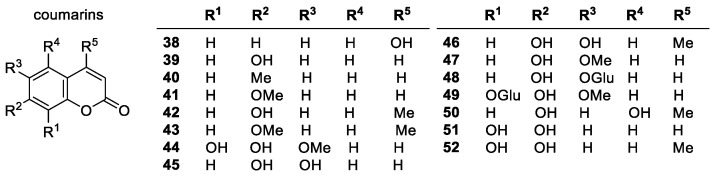
Structure of phenolic antioxidants with a coumarin core.

Coumarins are analogues of 2*H*-1-benzopyran-2-one (coumarin) and are identified by their lactone-based bicyclic structure. Their highly conjugated system and phenolic OH groups make them strong antioxidants including both natural and synthetic coumarins (Figure 5) [95,96,97,98]. The antioxidant activity of coumarins with two hydroxyl groups is the greatest, and those with a single OH group were much less active. Coumarins with no OH-group were inactive in all cases. Coumarins with substitution on their hydroxyl groups, such as herniarin (**41**), exhibited decreased activity. The important structural features of coumarins are the conjugated aromatic electron system, which allows for effective stabilization of radical products [99].

##### Stilbenes

**Figure 6:**
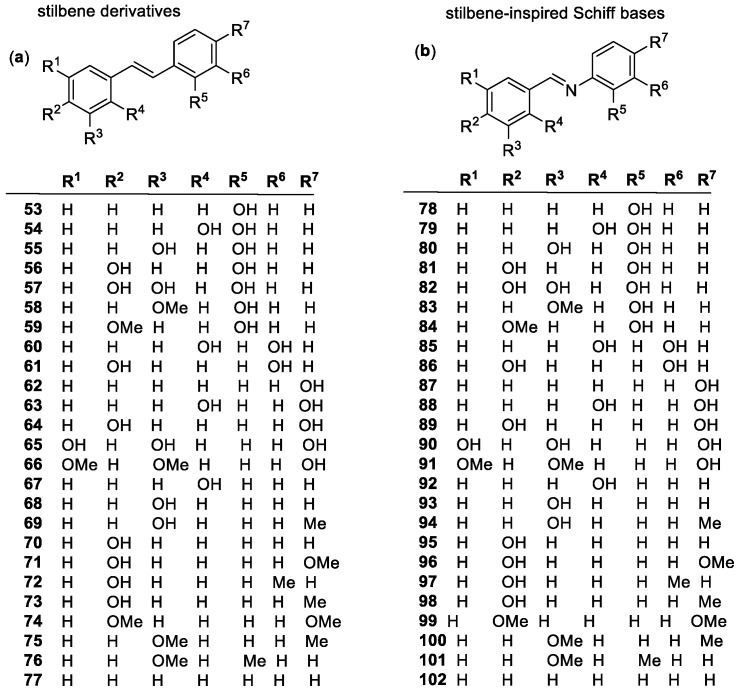
The structure of stilbene derivatives (**a**) and stilbene-inspired Schiff-bases (**b**) with reported antioxidant activity.

Stilbenes are derivatives of (*E*) and (*Z*) 1,2-diphenylethene (stilbene), having a basic structure in which two phenyl groups are connected by an ethene bridge (Figure 6a). The (*E*) isomer is the more common due to its higher thermodynamic stability. The most common stilbene-based antioxidant is resveratrol (**65**), with a 3, 5, 4′ hydroxyl substitution pattern. The polyphenolic nature of resveratrol is the source of its antioxidant activity, aided by the ethene bridge allowing conjugation between the two phenyl rings. The replacement of the bridge by a -CH_2_-CH_2_- or CH_2_ unit significantly lowers antioxidant activity, showing the importance of conjugation [100]. Other substitution patterns have been tested, and it was found that the location of the OH groups is extremely important. The 4′ location has high physiological importance [101], and even a simple 4,4′ substitution (**64**) was found to have a significantly higher antioxidant effect in cellular systems than that of resveratrol [102]. Studies with other stilbene derivatives found that methoxycarbonyl groups decreased antioxidant activity, and that the dimer of resveratrol (containing 4 phenyl and 5 OH groups) has higher antioxidant activity, reiterating the importance of the number of hydroxyl groups [103]. Resveratrol has also been shown to have a positive impact on ROS levels in brain mitochondria and may be an important protector of mitochondrial systems [104].

Stilbene-inspired Schiff bases or imines (Figure 6b) (**78**–**102**) also tend to show a higher, or at least similar, antioxidant activity than that of resveratrol. The more hydroxyl groups added to the derivative, the higher the DPPH scavenging activity is [105]. Although the 4′-OH is the most important for resveratrol, a single 2′-OH substitution (**78**) shows better DPPH scavenging results in Schiff bases. The *meta* position 3′ negatively affected antioxidant activity, lowering DPPH scavenging activities by a factor of 20, when comparing a 2′,2-dihydroxyl (**79**) to a 3′,2-dihydroxyl (**80**) [106].

##### Vitamin E Group

**Figure 7:**
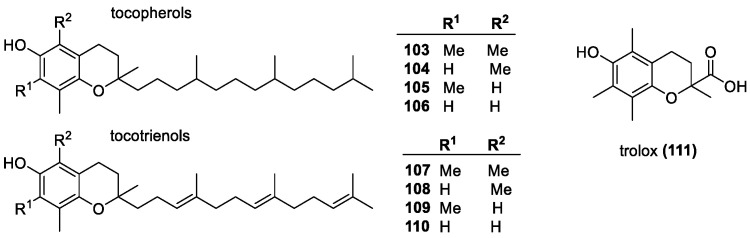
The structure of vitamin E-related antioxidants, including trolox a commonly applied reference compound.

The vitamin E family (Figure 7), named for the common name of α-tocopherol (**103**), containing tocopherols (**103**–**106**) and tocotrienols (**107**–**110**), collectively tocochromals, are phenolic antioxidants with a bicyclic chromanol ring structure and a long C_13_ tail. There are four derivatives in each group, named from α to δ, and corresponding names of each group feature the same substitution pattern, the only difference being in the carbon tail. In tocotrienols, the tail is an isoprenoid chain with double bonds, while in tocopherols, the chain is fully saturated. Their antioxidant mechanism is based on the phenolic hydroxyl group on the chromanol ring, which allows for the HAT mechanism and resonance stabilization across the conjugated ring, as well as the adjacent oxygen on the second ring. Every vitamin E derivative has the phenolic OH in the C6 position, with various substitutions on the aromatic side of the chromanol ring. They also have a methyl group at the 8 position, with α-tocopherols having a fully methylated ring, β-tocopherols having an additional methyl at the 5-position, γ-tocopherols at the 7 position, and δ-tocopherols only having a single one at the 8 position. These compounds are well-known as singlet oxygen and other ROS scavengers, especially in lipid environment due to their hydrophobicity.

α-Tocopherol has often been used to inhibit lipid peroxidation [107,108]. The hydrophilic variant of α-tocopherol, trolox (**111**), is also used as a standard in trolox equivalent antioxidant capacity (TEAC) assays and often used to gauge antioxidant activity as well [56]. ORAC assays show that the δ-tocochromanols (**106**, **110**) have the highest activity. The triene chain improves activity; however, the saturated lipophilic tail does not affect in vitro radical scavenging. Likely, its role is to anchor the compounds to cell membranes. Other assays, such as the DPPH and FRAP suggest that α-tocochromanols (**104**, **108**) indicate an increase in scavenging ability correlating with the degree of methylation in the *ortho*-position to the OH group. The removal of the methyl groups leads to higher activity in assays likely due to reduced steric hindrance. Some studies have observed the opposite, with EDGs in the *ortho*- and *para*-positions leading to enhanced stability and reactivity against peroxyl radicals. The isoprenoid-like tail allows for vitamin E derivatives to be lipid soluble, and they often interact with cell membranes. It results in better membrane penetration and inhibition of lipid peroxidation and for non-antioxidant functions, increased membrane stability. They can anchor themselves to the lipid bilayer with the chromanol ring at the surface to foster the easier regeneration of the phenol form from the radical [109,110,111,112,113,114,115,116,117,118,119,120,121,122,123]. As mentioned above, vitamin E can interact with vitamin C at the surface of lipid bilayers, allowing for the hydrophilic chromanol ring to be regenerated by ascorbic acid to its original radical scavenging ability, while creating the more stable and less reactive ascorbyl radical [75,81,82,83,84].

##### Curcumins

**Figure 8:**
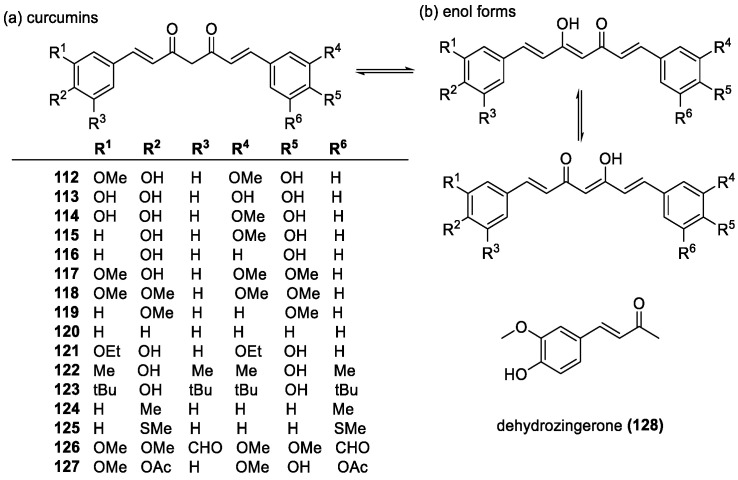
The structure of selected curcumin-based antioxidants.

Linear diarylheptanoids, also called curcuminoids, are plant metabolites consisting of two aromatic rings connected by C_7_-bridge with 3,5-dicarbonyl and 1,6-diene groups (Figure 8). Curcumin (**112**), found in turmeric, is the yellow pigment giving turmeric its distinct color. They are known for their anti-inflammatory and anti-cancer properties, and their potent antioxidant activity [124,125]. By examining analogues of curcumin, it was found that having a *para*-hydroxyl group in the two phenyl rings was enough to create an antioxidant effect [126]. Electron donating groups, such as methoxy also play an important part in curcuminoid antioxidant activity, with *ortho*-substituted variants showing enhanced stability of the radical form [127]. Even methyl and ethoxy groups in place of the methoxy groups (**121**, **122**) were found to have similar DPPH results as that of curcumin and showed even higher inhibition of lipid peroxidation. Reduction of the C=C double bonds in the bridge did not affect the inhibition of lipid peroxidation, and increased the total DPPH score, though resulted in much slower scavenging [126]. Another study compared dimethoxycurcumin and curcumin, and found that the methylene groups were inconsequential to the activity [128]. The dicarbonyl group is important regarding the antioxidant activity, as a hydrogen can be abstracted in either enol forms (Figure 8b) [129], or potentially from the active CH_2_ group in between the carbonyls. A study on dehydrozingerone (**128**), or “half-curcumin”, containing one phenolic OH and one carbonyl group found that it does not react under certain conditions that curcumin does, and even when it does, the reaction rates are much slower. This difference highlights the importance of the enolic forms and that H-abstraction from the phenolic OH groups accounts for only 15% of the total radical scavenging [130].

##### Lignans

**Figure 9:**
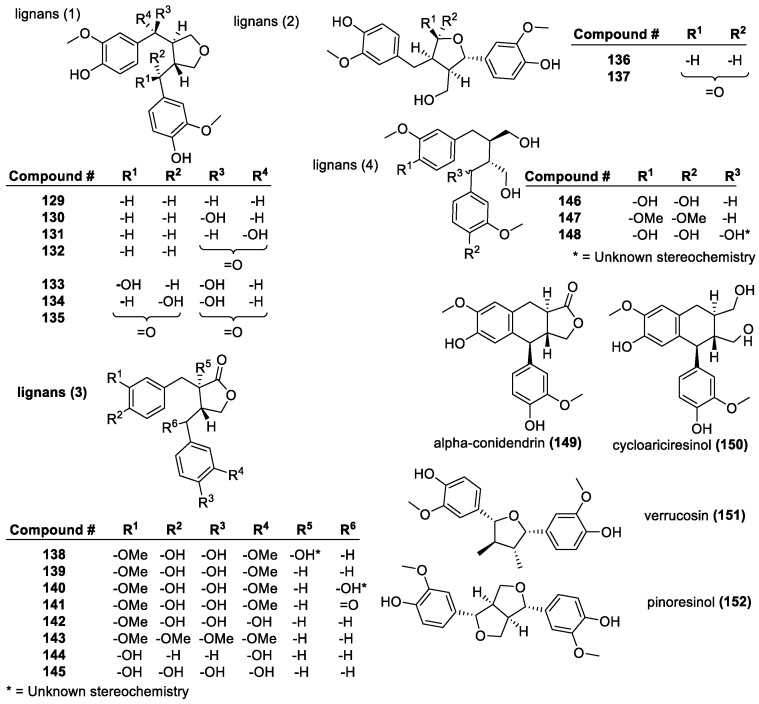
The structure of selected lignan-based antioxidants.

Lignans are a large and diverse group of polyphenols, produced in plants via the oxidative dimerization of two phenylpropanoids. The three most common lignans are hydroxymatairesinol (**140**), matairesinol (**139**) and secoisolariciresinol (**146**) (Figure 9), although synthetic lignans have also been made [131]. While the overall structure of lignans varies, there are specific features that correlate to their antioxidant activity. It was found that the catechol structure in **145** led to the highest antioxidant activity with a DPPH score (DPPH/AH = stoichiometric factor at EC_50_, AH = antioxidant) of 4.7, compared to matairesinol (**138**) with 3,3′-OMe groups and a score of 2.9. Compounds **139** and **146** also have similar structures, as do **135** and **136**, plus **140** and **148**, differing in that the closed butyrolactone ring is being replaced with an open butanediol unit. Other compounds, such as **150** and **146,** also differ in this regard, with **150** being closed and **146** being open. Comparing all of these, systems with open diol are significantly more active and efficient than the cyclic version. The molecule has a single *meta*-OH group on each ring (**144**), which eliminates any significant antioxidant effect, since the *meta*-position is less stable in radical form. The fully methylated forms of **147** and **159** also show no activity, as expected since there are no phenolic hydrogens to be abstracted [39,132]. This is largely in-line with structural features of antioxidants, especially since the catechol hydroxyl substitution greatly improves antioxidant activity. The addition of oxygen-containing substituents to the R^6^ position on **138**–**145** was also found to lower activity; thus, having a free benzylic position without any substituents is required for antioxidant activity, with hydroxy ketones being significantly more effective than a hydroxyl alone. In addition, the stereochemistry of a hydroxyl group on the carbon adjacent to the ketone carbon (R^5^ position in **138**–**145**) on the lactone ring can also affect the stability and activity of the molecule, with *cis*-hydroxyl being more stable and having higher activity than the *trans*-isomer [133]. Comparing matairesinol (**138**) to lignans with lactone revealed that the esters were more potent antioxidants than the lactone variants. Increasing the degree of oxidation had a negative effect on the antioxidant activity of lignans as well, with hydroxyl or carbonyl groups in the benzylic position leading to decreased potency. This effect is remarkable with diketones [134,135]. The position and proximity of the phenyl groups also has an impact on the scavenging ability of lignans. When fewer bonds separate the phenyl rings on either side of the structure, the scavenging abilities are lower [136].

##### Flavonoids

**Figure 10:**
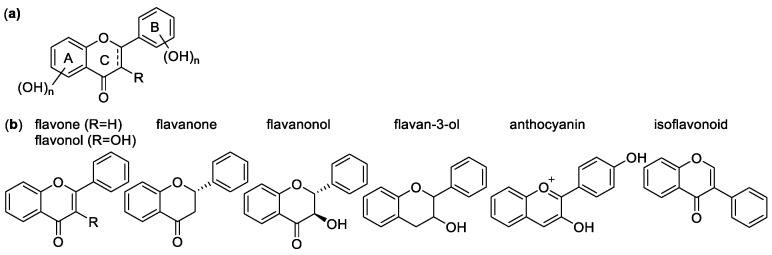
The generic structure (**a**) and major classes of flavonoids(**b**).

**Figure 11:**
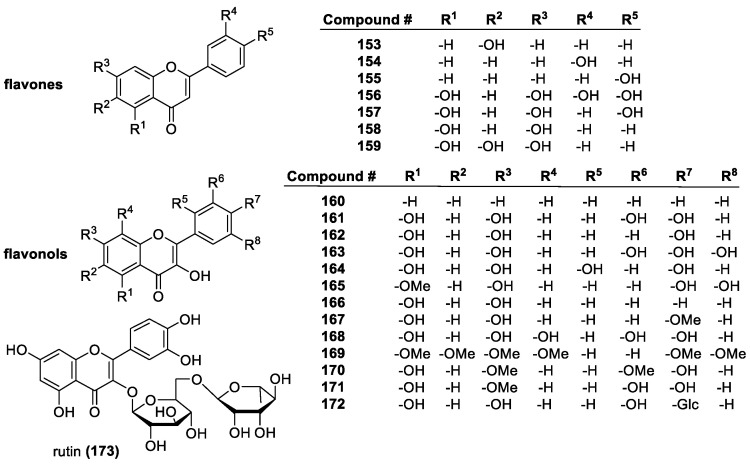
Representative examples of flavones and flavonols with antioxidant effect.

**Figure 12:**
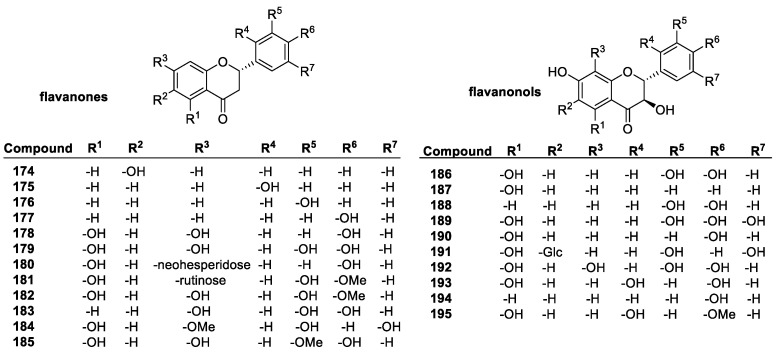
Representative examples of flavanones and flavanonols with antioxidant effect.

**Figure 13:**
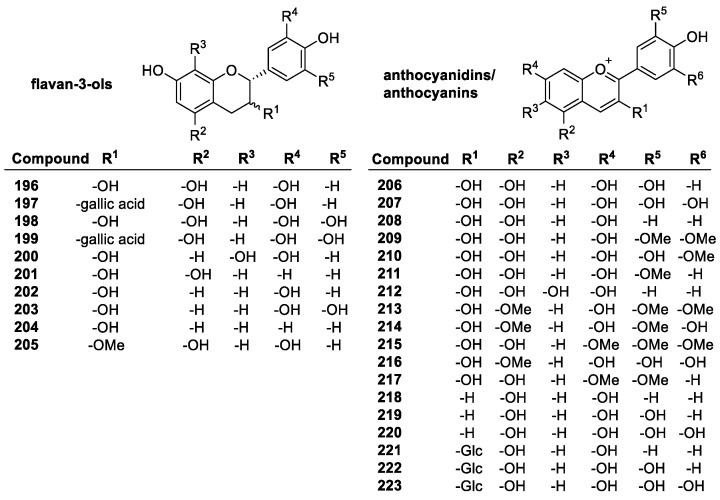
Representative examples of flavan-3-ols and anthocyanidins/anthocyanins with antioxidant effect.

**Figure 14:**
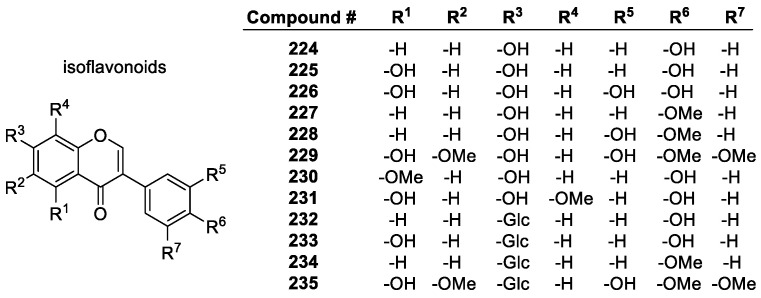
Representative examples of isoflavonoids with antioxidant effect.

Flavonoids are a diverse group of plant-based polyphenols with a C_6_-C_3_-C_6_ three- ring core [45,96]. The two C_6_ rings, A and B, are aromatic with varying degrees of hydroxylation (Figure 10a). The C2-C3 bond in the O-containing ring is often a C=C double bond [137]. There are several subclasses of flavonoids, all with distinct characteristics, and over 6000 individual compounds are known [138]. Among the main subclasses are flavanones, flavan-3-ols, flavonols, isoflavonoids, anthocyanins and anthocyanidins (Figure 10b) [139]. Representative examples of these groups are depicted in Figure 11, Figure 12, Figure 13 and Figure 14.

The main antioxidant mechanism of flavonoids is the HAT, with the hydrogen originating from the phenolic OH groups. Thus, OH groups are extremely important to flavonoid antioxidant activity, and there is a linear correlation between number of free hydroxyls and activity [140,141]. In an ORAC assay of flavones with a single hydroxyl group at 3, 6, 3′, or 4′-positions have slope values correlating to radical scavenging, of 0.384, 0.361, 0.212, and 0.603 ORAC activity, respectively, which is significantly lower than that (2.671) of kaempferol (**162**), with four active hydroxyl groups. For flavanones, with 6, 2′, 3′, 4′, or 7′ single hydroxyl group, the ORAC slope values are 1.361, 0.297, 0.212, 0.249, and 0.129, respectively, compared to the three-OH group-containing naringenin with 2.669 [142].

Although more hydroxyl groups increase antioxidant activity, there are limitations to this. Studies have shown that a third hydroxyl group in the B-ring does not increase antioxidant efficiency. Quercetin (**161**) and myricetin (**163**) have almost identical structures, but myricetin has an additional hydroxyl group at position 5′, and lower TEAC activity. Other methods, however, e.g., DPPH, electron spin resonance (ESR) spectroscopy, and the FCR total phenolic content assays, show higher activity for myricetin [143].

One of the most important structural patterns for the antioxidant activity of flavonoids is the 3′,4′ B-ring OH substitution. Referred to as a catechol group after the compound of the same name, this pattern appears to be one of the best indicators of antioxidant activity [144]. Another factor is the level of conjugation in the system. Flavonoids with a conjugated C-ring tend to have higher antioxidant activity, as demonstrated with quercetin and taxifolin (**186**), which have the same substitution, but quercetin contains a conjugated C-ring, and exhibits more than double the antioxidant activity of taxifolin (1.9 vs. 4.7).

Common flavones include luteolin (**156**), apigenin (**157**), chrysin (**158**), and their derivatives. Although they possess a conjugated system, their activity tends to be lower compared to other flavonoids, particularly flavonols. Since they lack the 3-OH group, they lose a potential active site. The catechol substitution does improve activity, especially that of luteolin (**156**) and rutin (**173**), the latter of which is a glycoside of quercetin with the disaccharide rutinose. They have trolox equivalents of 2.1 and 2.4. The flavone with one of the highest activities, baicalein (2.56 trolox equivalent), is a bit anomalous. The B-ring is completely unsubstituted, but it has a distinctive 5,6,7-hydroxyl pattern on the A-ring, which overcomes the lack of conjugation and B-ring OH groups [145].

Isoflavones are isomers of flavones, with the B-ring in the 3-position instead of the 2-position of the C ring. They tend to have similar radical scavenging activity to the corresponding flavones with regards to the same hydroxyl group position and sugar moiety, showing that the position of the B-ring has little consequence in antioxidant activity [146].

Common flavonols include quercetin (**161**), kaempferol (**162**), myricetin (**163**), and morin (**164**). Their highly conjugated structure allows for good resonance stabilization of radicals, and they tend to have a higher activity than the similar flavones. The 3-hydroxyl group and conjugated system give them an advantage over other flavonoids, particularly flavones, which have a similar ring system, but lack the 3-hydroxyl group. Esterifying the 3-OH, as with rutin, can also lower activity, to 2.3 trolox equivalents from 4.6 [145].

The flavan-3-ols e.g., catechin (**196**, *trans*) and epicatechin (**196**, *cis*) have the same number of hydroxyl groups as quercetin, and in the same positions, but the antioxidant activity is much lower at 2.4 and 2.5 trolox equivalents, respectively, compared to 4.6. due to the lack of conjugation from the 2–3 double bond. By adding an additional hydroxyl group in the B ring to form epigallocatechin (**199**), the antioxidant activity increases to 3.82 trolox equivalents. Further modifications to catechin derivatives include esterification of the 3-hydroxyl group with gallate, resulting in epicatechin gallate and epigallocatechin gallate, with a total of 7 and 8 hydroxyl groups, bringing their activity to 4.93 and 4.75 trolox equivalents, respectively [142].

Common flavanones, such as naringenin (**178**), naringin (**180**), hesperidin (**181**), and eriodictyol (**179**), tend to show lower antioxidant activity than other flavonoids, mostly due to the lack of conjugated C-rings. This decreases the resonance stabilization for radical products, as well as the number of active sites. For example, quercetin and taxifolin only differ by the lack of the C2-C3 double bond in ring C, but the activity of taxifolin is 1.9 trolox equivalents, compared to 4.7 trolox equivalents of quercetin [137].

Anthocyanidins and anthocyanins are natural plant pigments characterized by their ionic structure. Anthocyanins are glucosides of anthocyanidins, containing a sugar unit most commonly in the 3- or 4-position. Their aglycons are generally more efficient antioxidants, but this is not always the case. They are very good antioxidants, known to trap lipid peroxyl radicals effectively in addition to other ROS. This is due to the unusual oxonium ion structure in the C-ring, as well as their conjugated system that stabilizes radical products due to the possible delocalization of electrons [147]. Compared to other antioxidants in a DPPH assay, the anthocyanins and their aglycons had similar antioxidant activity to common phenolics. According to this study [148], cyanidin (**206**) had a score of 33% radicals scavenged, delphinidin (**207**) 42%, pelargondin (**208**) 31%, malvidin (**209**) 24%, petunidin (**210**) 10%, and peonidin (**211**) 33%. Delphinidin has a 3′, 4′, 5′ hydroxyl substitution, giving it the highest activity, but the effect of the extra OH group was not noticed elsewhere. Malvidin has MeO groups at 3′ and 5′, giving it lower activity compared to the others. The one with the lowest activity, petunidin, has a 3′-MeO group and a 5′-OH group. While the conjugation and resonance stability help the activity of anthocyanins, the OH groups are still the most important factor in determining how effective they are as antioxidants. Glycosylation of these compounds had varying effects, ranging from insignificant to large. The 3-glucose substitution had little effect on the activity of delphinidin, malvidin, and cyanidin, but petunidin activity increased from 10 to 23%, while peonidin and pelargonidin had lower activity. Other sugar substitutions had a mildly negative effect on activity [149].

##### Tannins

**Figure 15:**
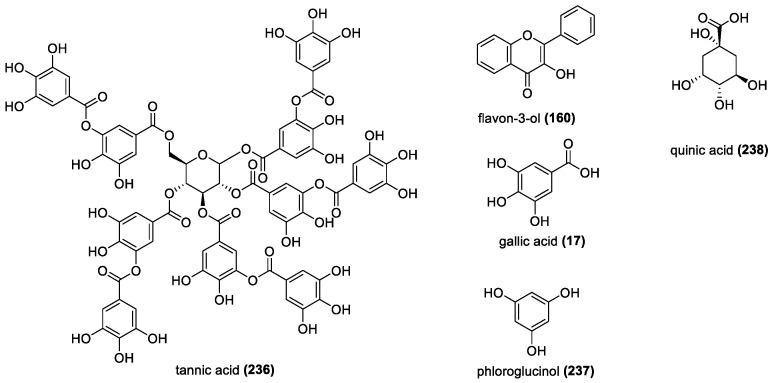
Structure of tannic acid and the most common units that form tannin scaffolds.

Tannins are high molecular weight polyphenols that contain many phenolic OH groups and are typically divided into three common groups: condensed tannins (proanthocyanidins), hydrolysable tannins, and phlorotannins. Proanthocyanidins are polymers of flavon-3-ols, such as the previously discussed catechin derivatives, and hydrolysable tannins contain a core consisting of sugars or polyols, several hydroxyl groups, and are esterified by gallic acid or hexahydroxydiphenic acid (Figure 15) [149].

Due to the high number of phenolic OH groups, tannins are strong antioxidants; 10–30 times more effective than simple phenols or trolox [150]. While smaller antioxidants, especially flavonoids, depend more on the number and location of hydroxyl groups for their activities and favor small substituents, tannins tend to do better with galloyl substitutions. For example, the tannin with the highest antioxidant activity in one assay, the tetrameric ellagitannin sanguiin H-11, has a molecular weight of 952 and is an ellagitannin-based oligomer. This is a generic trend as dimeric and trimeric configurations showed the highest activity for all tannins, and comparisons between larger and smaller compounds showed that only the number of galloyl groups was important. [151]. It was also found that the galloyl group was necessary for tannins to be effective, as even dihydroxyphenol group-based tannins showed no scavenging [152].

Quinic acid-based tannins also follow the same trend that more galloyl groups increase activity. Quinic acid (**238**) has no phenolic OH groups, and therefore no activity, but adding galloyl groups increases activity significantly. The position was found to be important with the digalloyl groups, but compounds with either 3 or 4 attached galloyl groups all had similar activity, with the tetra being higher. The 3-position from the quinic acid ring provides the best activity, with the 3,5-digalloyl derivative having the best effect among the disubstituted group. All of the highest performing esters had the 3,5-positions substituted, and all outperformed gallic acid, and even known scavenger epicatechin in the DPPH assay. In a liposome peroxidation assay, all of the esters, except the 3-monoester, outperformed gallic acid by a significant margin, although epicatechin outperformed them all [153]. This could be due to steric effects caused by the bulkiness of the tetra-galloyl groups, allowing epicatechin to move more freely through membranes.

### 3.2. NH-Containing Antioxidants

#### 3.2.1. Heterocyclic Compounds

##### Melatonin

**Figure 16:**
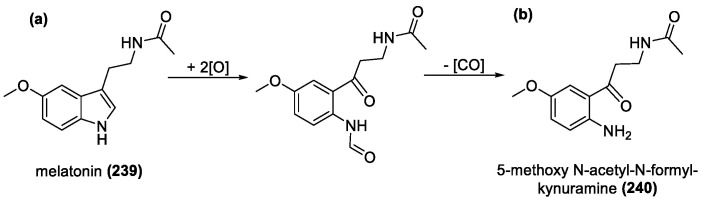
Melatonin (**a**) and its reaction to form 5-methoxy N-acetyl-N-formylkynuramine (**b**).

Melatonin (**239**) (Figure 16) is an amphiphilic triptamine derivative, mostly known for its role in promoting sleep and is available as a sleep supplement in many countries. It contains an electron donating methoxy group at the 5-position, and an acetyl group attached to triptamine NH_2_ group at the 8-position. The methoxy group appears beneficial, if not necessary to antioxidant ability of melatonin. It does prevent the full semi-quinone radical interactions, similar to vitamins and thiols, and aids improved resonance stability of the radicals. The acetyl side chain has also been implicated to improve lipophilicity which promotes onsite binding in cellular membranes to prevent oxidative damage [154]. It is a known scavenger for both ROS/RNS and has been shown to be twice as effective at scavenging peroxyl radicals than vitamin E [155]. Melatonin can prevent oxidative stress in mitochondria [154,156,157,158], which makes it a prime target for treatment of mitochondrial disorders. It is relatively resistant to oxidation but does react with highly reactive radicals such as hydroxyl radicals or metal complexes, making it selective. When compared to glutathione, it is more potent with regards to hydroxyl radical scavenging, with IC_50_ value of 21 μM compared to 286 μM. It works solely via the ET mechanism and when oxidized, forms an indolyl intermediate which further reacts with another hydroxyl radical to form 5-methoxy-N-acetyl-N-formylkynuramine (**241**), which fully reduces the two initial radicals. The new product has additional iron chelating and antioxidant effects as well. Modifications of the indole ring can increase antioxidant activity, as observed when testing their protective effects on human cells [159].

##### Pyrroles

**Figure 17:**
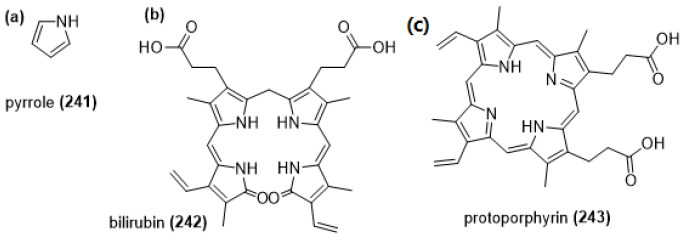
Structures of pyrrole (**a**), bilirubin (**b**), and protoporphyrin (**c**).

Pyrrole (**241**) and its derivatives are nitrogen-containing heterocyclic aromatic compounds, with a functional -NH group as their active pharmacophore. Similar to the phenolic antioxidants, the mechanism for the activity of these compounds is HAT- or ET-based, with the hydrogen in the aromatic NH group acting as the donor. The conjugation in the aromatic ring of pyrroles allows for the radical to be resonance-stabilized. The most common pyrroles to exhibit antioxidant activity are condensed tetrapyrroles such as bile pigments, e.g., bilirubin (**242**) and protoporphyrin (**243**) (Figure 17) [160].

Bilirubin (BR) is a highly conjugated tetrapyrrole and it is well-known as the end product of heme catabolism and a principle yellow bile pigment, a jaundice-causing toxin. It has shown significant antioxidant activity due to its conjugated nature [161,162]. It is one of the most abundant antioxidants in mammalian tissues, and in vitro studies found that it can prevent lipid peroxidation, especially Cu^2+^-mediated oxidation of LDL. It can also work synergistically with vitamin E to inhibit peroxidation of liposomes, and potentially scavenge hypochlorous acid under physiological conditions. Compared to other endogenous antioxidants, it is far more effective at peroxyl scavenging than thiols or ascorbate, and although more effective at superoxide scavenging than vitamin E, it is less so than ascorbate. Frequently bound to serum albumin, and albumin-BR complexes being effective antioxidants as well, it can also protect albumin oxidation. Higher BR plasma concentration has been linked to higher antioxidant activity in humans [162].

#### 3.2.2. Aniline Derivatives

**Figure 18:**
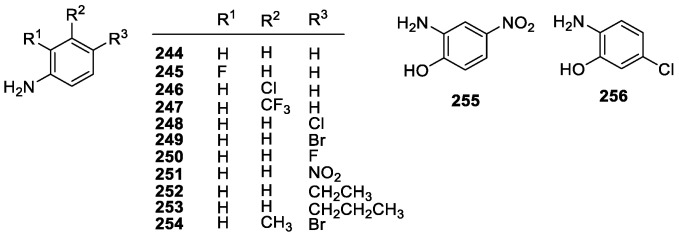
Structure of anilines studied in a comparative study with phenols.

Aniline, or amino benzene, is the simplest aromatic amine. It is a weak base and a relatively poor nucleophile, but due to being electron rich, it undergoes electrophilic aromatic substitution very rapidly and oxidizes easily. Anilines are similar in structure to phenols, having an NH_2_ group instead of OH. Studies on the antioxidant effects of aniline derivatives are relatively sparse, however. In a recent phenols-anilines comparative study it was found that anilines have comparable scavenging abilities to phenols, given the large range of activities seen in both groups (Figure 18), with some being better than others. The general trend was that EDGs could increase the overall activity, and EW groups could lower activity, which has been seen in other studies. It was also found that they tend to act under HAT mechanisms, but there is evidence for strong SET activity under certain conditions [62].

#### 3.2.3. Imines and Hydrazones

**Figure 19:**
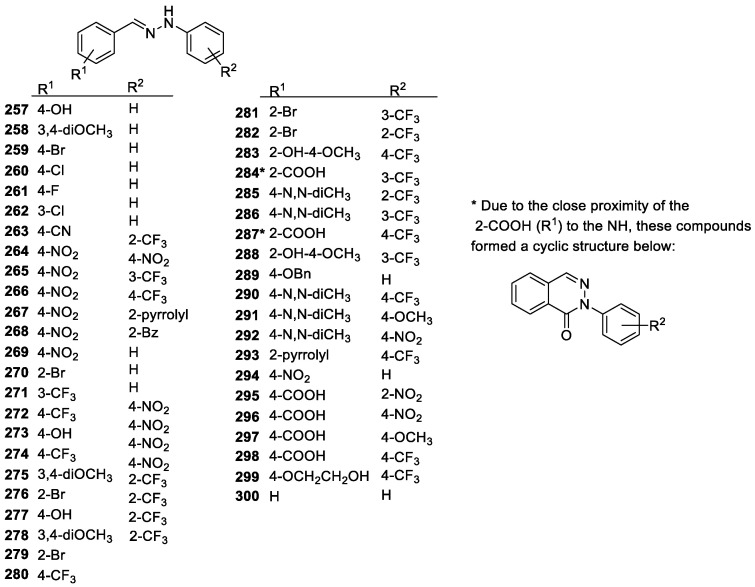
Representative structures of diarylhydrazone antioxidants.

Imines/Schiff-bases (Figure 6b, see above) and hydrazones (Figure 19) are two related compound groups similar to carbonyls but contain a C=N double bond instead of a C=O bond. Imines are obtained from the reaction of a carbonyl compound with primary amines, while using hydrazines produces hydrazones. When containing two phenyl rings, these derivatives resemble the structure of resveratrol. They are effective antioxidants as well, although the activity of imines is largely dependent on the presence of phenolic hydroxyl groups [106]. Hydrazones, however, are true NH-based antioxidants even without the presence of hydroxyl groups [163]. Other studies on diaryl hydrazones report similar or higher scavenging abilities than that of resveratrol and other reference antioxidants and are potential therapeutic agents. In addition, the unsubstituted diaryl hydrazone had moderate activity, indicating that while NH is a potential pharmacophore, the substituent effects are important to improve its scavenging abilities. This is likely due to changes in the BDE of the NH bond caused by the substituents [164]. Several hydrazone derivatives have been tested, including a melatonin-based one, and most tend to show good antioxidant activity [165,166]. As for the substituent effect, it was found that carboxylic acid and NO_2_ groups had the greatest impact on overall activity, both of which increase the delocalization of the electrons in the compound allowing for greater stabilization of the radical. Hydrazones with partially or perfluorinated phenyl rings were applied for the inhibition of the CK2 enzyme, and as a multitarget agent showed excellent antioxidant effect [167]. These fluorinated hydrazones were found to be highly active in mitochondrial antioxidant therapy to alleviate the symptoms of preeclampsia as well [168].

#### 3.2.4. Betalains

**Figure 20:**
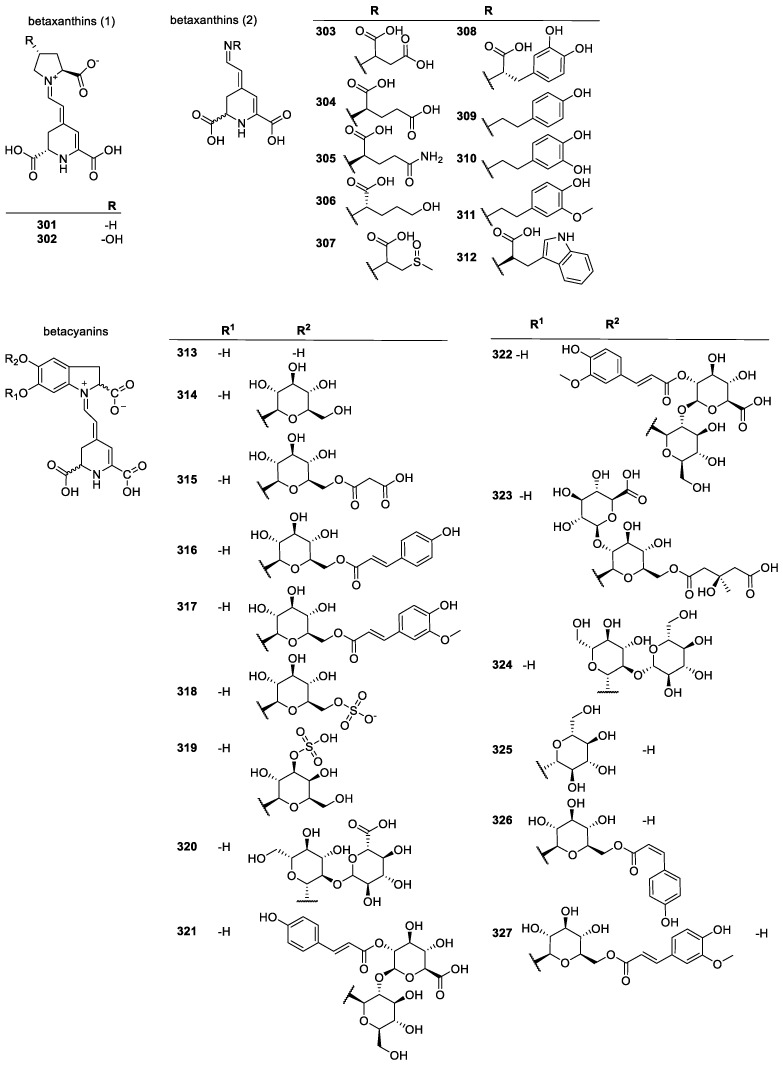
Structures of betaxanthins and betacyanins.

Betalains are tyrosine-based pigments, replacing anthocyanins in certain plants. They have two subcategories, betacyanins and betaxanthins. Betacyanins are derivatives of betanidin (**313**), and betaxanthins are derivatives of betalamic acid with various amines and amino acids (Figure 20) [169,170]. Plants with high betalain content have high antioxidant activity, exceeding that of catechin (**196**) [29]; both in radical scavenging and lipid peroxidation inhibition, compared to other plants with low betalain content [25,171].

Betacyanins and betaxanthins have the same basic core: a piperidine ring containing two carboxyl groups in 2,5-positions. There is at least one C=C double bond in the ring, adjacent to one of the carboxyl groups. A conjugated imine side chain is located in *para* position to the nitrogen. In betacyanins, there is an iminium cation with a carboxylate anion adjacent to the imine at the 2-position, and a phenolic ring adjacent at the 4- and 5-positions. In betacyanidins, this is a diphenol, and in betacyanins, one of the phenols is a glucoside ester. Betaxanthins contain an imine motif, which can either be part of the proline ring, or a side chain. It lacks the bicyclic structure of the betacyanidins and contains no phenolic OH. Glycosylation increases stability in cyanins, although additional glucose groups in certain cases can decrease it. Steric effects improve stability, with the 6-O-esters being more stable than the 5-O-esters [41,172,173,174].

A study on the SARs of betalains found that a higher number of H-donating groups, hydroxyl and NH, increases the activity, with the 5-OH position leading to the highest activity likely due to greater H-donating capability. Increasing the glycosylation of the molecules led to lower activity [175]. Although betalains tend to be hydrophilic, the two most known betalains, betanin and indicaxanthin are known to bind to human lipoproteins, specifically LDL, and prevent the onset of oxidation. Indicaxanthin had a stronger effect, indicating that its mechanism may be more effective. Unlike many antioxidants, usually they do not show any pro-oxidant effect over large concentration range [176,177].

A different study also noted that the affinity of betacyanins to liposomes is likely caused by its cation being attracted to the negative charges of membranes [170]. Betacyanins are glucosides; studies of their aglycones (betacyanidins) have shown that the sugar-free variants tend to degrade faster and are less effective at liposome bonding [170]. Betacyanins are also more stable and effective scavengers than betaxanthins, due to the phenolic groups in the former, whereas the latter have no phenolics and rely on their conjugated π-systems for electron abstraction [178].

### 3.3. SH-Containing Antioxidants

#### 3.3.1. Glutathione

**Figure 21:**
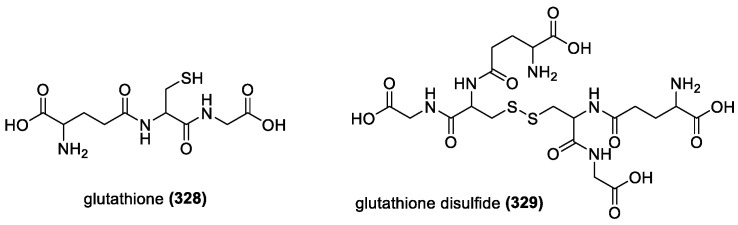
Structure of glutathione in its monomer sulfhydryl and disulfide forms.

One of the most important endogenous antioxidants, glutathione (**328**), is among the earliest discovered and most studied examples (Figure 21).

Glutathione (**328**) (GSH) is the active form of the compound that reduces peroxides in a reaction catalyzed by a selenium-enzyme glutathione peroxidase, while being converted to the oxidized form glutathione disulfide (**329**) (GSSG). Reduction of the glutathione disulfide occurs via the enzyme glutathione reductase, which breaks the disulfide bond of the GSSG by oxidizing NADPH into NADP^+^ [63]. It is also shown to act in conjunction with vitamin E in preventing lipoperoxidation, with GSH taking a preventative role in low concentrations of vitamin E [179].

#### 3.3.2. Cysteamine and Penicillamine

**Figure 22:**
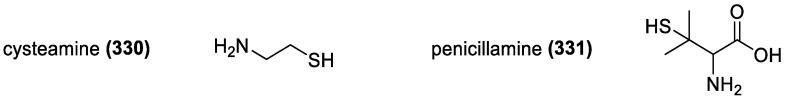
Structure of cysteamine and penicillamine.

Cysteamine (**330**) contains both amino and thiol groups (Figure 22). It has been shown to reduce lipoperoxidation and increase glutathione peroxidase activity, indicating it is a potent ROS scavenger. Since it is often lipid-bound, it works to prevent peroxidation and reduce the number of potential products. It can also chelate iron ions within lipids, thereby reducing their harmful effects [180]. It also works alone, being an effective antioxidant reducing the onset of autooxidation of cytotoxins [181]. Penicillamine (**331**) (Figure 22) is used as a copper-chelating drug and has antioxidant activity. It has been effective in preventing the formation of ROS in human cells [182] and was found to use both the amino and thiol groups to prevent the onset of autooxidation. However, the inhibition of either SH or NH_2_ limited its antioxidant potential [181].

### 3.4. Isoprenoid Antioxidants

#### 3.4.1. Carotenoids and Xanthophylls

**Figure 23:**
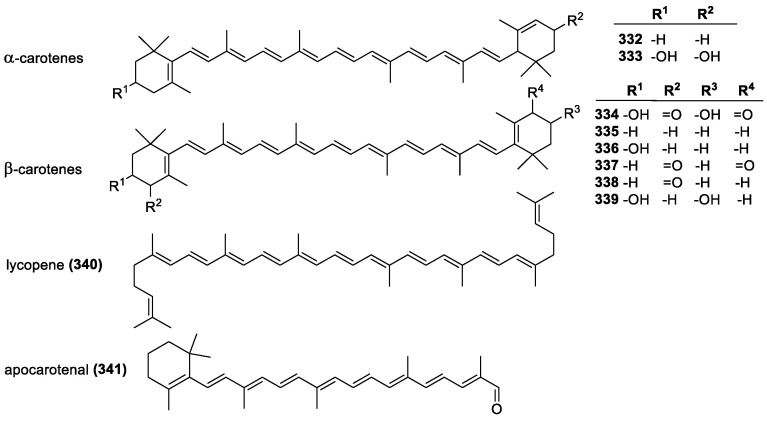
Representative examples of carotenoids.

Terpenoids, also called isoprenoids, are derivatives of isoprene and terpene polymers (Figure 23). Common examples are carotenoids and xanthophylls. Common structural features of carotenoids and xanthophylls are the isoprenyl chain connecting two cyclohexene rings, often with a 1,3,3-trimethyl substitution pattern. The chain conjugation ensures that π-electrons are delocalized across the entire system, giving them distinctive reactivities. Carotenoids are hydrocarbons, while xanthophylls (**333**, **334**, **336**, **339**) contain oxygen. Another common isoprenoid is lycopene, an acyclic isoprenoid [66,183]. Although commonly seen as all-*trans* isomers, various *cis* isomers are also known.

Carotenoids are known lipid antioxidants, and although they do not contain active groups seen in other antioxidants, they are effective quenchers of singlet oxygen and peroxyls [184,185]. This activity is known to root from their highly conjugated structure, with nine or more conjugated double bonds giving the highest activity [186]. The chain enhances the stabilization of carotenoid radicals when a hydrogen is abstracted [187]. The proposed mechanism is based on the idea that the carotenoids bind directly with the radical forming a carotenoid-peroxyl radical, which can reduce the concentration of peroxyl radicals. This mechanism works with all carotenoids and some other retinoids as well since their antioxidant properties are based on the high degree of conjugation [188]. Other proposed mechanisms are the ET and HAT, although the HAT is much less likely. The specific pathway depends on the type of radicals and carotenoids involved [189,190,191]. Oxygen pressure (above 150 Torr) results in lower antioxidant activity and the potential to become pro-oxidant. The overall hierarchy is known to be approximately lycopene (**340**) > beta-carotene (**335**) > zeaxanthin (**339**) > lutein (**333**) > echinenone (**338**) >> canthaxanthin (**337**) ~ apocarotenal (**341**) > astaxanthin (**334**) (Figure 23) [192]. This can vary depending on the type of quenched radical species however, beta-carotene reacting faster with chloroform [193]. Opening the beta-ionone ring can also significantly improve quenching abilities, with lycopene being the most effective singlet oxygen quencher. The activity steadily decreases with closed or substituted rings [194].

Substitution and conjugation of the ring can also influence the activity. Alpha-carotene (**332**) and beta-carotene (**335**) differ only in one of their rings, which contains either an adjacent and conjugated-(beta-carotene) or a non-conjugated double bond (alpha-carotene). Accordingly, the activity of alpha-carotene is lower since there is one less conjugated bond to stabilize the radical [195]. Oxygen containing functional groups also influences activity. Carbonyl groups significantly lower activity by over 50%; a single OH group on one ring has a negligible effect and OH on both rings lowers activity moderately. This effect is due to the electron-withdrawing nature of the oxygen which reduces the electron density, and therefore reactivity, of the conjugated chain [196]. Singlet oxygen, peroxyl radicals, and hydroxide radicals all react differently with carotenoids, with beta-carotene being more reactive towards peroxyl radicals and singlet oxygen [197].

Carotenoid radicals can be recycled by interactions with other antioxidants, namely vitamins E and C. Similar to the previously mentioned regenerating interactions of vitamins C and E, beta-carotene radicals are able to interact with both. Beta-carotene has also been proposed to “repair” spent vitamin E molecules by reducing their radicals, and the resulting carotene radical is in turn reduced by vitamin C [198]. This synergistic interaction has been demonstrated in vitro and in vivo and shows that antioxidant activity is greatly improved when all three molecules are present [199,200]. Beta-carotene and vitamin E mixtures also work synergistically to reduce lipid peroxidation, with vitamin E acting as a protector of the carotene molecules allowing them to be consumed much more slowly and react more effectively [201]. The length of the carotenoid chain is approximately the same as a lipid-bilayer, meaning that the entire length of the molecule could be within a membrane and potentially trap radicals [187].

Carotenoids are specifically known to help with photo-protection in humans. Light exposure can cause destructive ROS to form, which can damage cells in the body. Since carotenoids absorb light in the blue and UV wavelengths, topical application and dietary ingestion of carotenoids have been shown to lessen the effects of sun burn. The dietary ingestion increased the level of lycopene and total carotenoids in the skin, allowing for them to absorb most of the energy from the sunlight to prevent damage [202].

Different carotenoids align themselves differently, depending on their end groups. Beta-carotene locks itself into the core of a lipid bilayer since it is a pure hydrocarbon and typically lies in parallel with the edges of a bilayer. Zeaxanthin and other xanthophylls have hydroxyls group on their rings, which allows the rings to anchor along the edges of the lipid bilayer, where the hydroxyls interact with aqueous solutions. Lutein, on the other hand, can freely rotate one of its rings, so it can anchor itself along the edges and lie in parallel to it. Thus, carotenoids that anchor along the edges are better at inhibiting lipid peroxidation [203], with the anchored xanthophylls also preventing oxygen from entering and strengthening the overall membrane [204,205].

#### 3.4.2. Retinoids and Vitamin A

**Figure 24:**
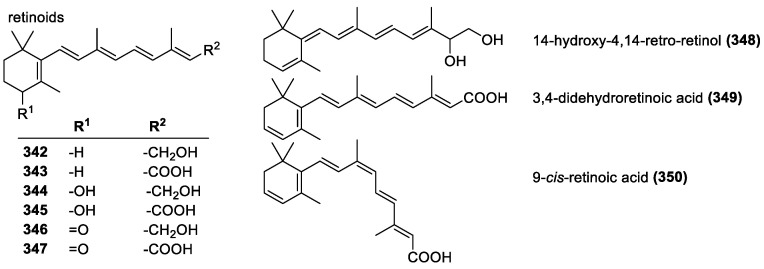
Structure of common natural retinoids.

Retinoids are similar to carotenoids, but they only contain one cyclohexene ring (Figure 24). Retinol (**342**), the most common in this group, has a hydroxyl group in place of the other ring found in carotenoids. Vitamin A, a group of retinoid compounds (retinol, retinyl esters, etc.) are essential for vision and retina health and also include the aldehyde retinal and provitamin A carotenoids. Beta-carotene is considered a pro-vitamin because although it is not a vitamin itself, it is used as a precursor in retinol biosynthesis.

Retinol (**342**) and its analogs are lipid-protecting antioxidants in vitro and in vivo [206,207]. Other studies, however, have shown that it may act as a pro-oxidant under certain conditions and increase oxidative stress [208]. Even under conditions favorable to antioxidant activity, it is highly–rate-limited and only a minor fraction of retinol is consumed in scavenging reactions. It is also highly likely to undergo self-oxidation at high oxygen pressures [209].

Many of the same structural features that give carotenoids their activity, namely the isoprenoid chain, are also in play with retinoids, but the mechanisms differ. Retinoids react by the addition of the radical to the cyclohexenyl ring, which in turn has a few ways to increase stability. These are direct additions of oxygen, the lipoperoxyl radical, or a unimolecular decomposition into an epoxide and alkoxyl radical after lipoperoxyl radical addition. Of these, only the double addition of the lipoperoxyl radical is effective at chain breaking; however, most retinol transforms to the ineffective epoxide form. With that, at low oxygen pressures (below 15 Torr), low concentrations, and high radical flux, it can be very effective [208].

### 3.5. Compounds with Combined Pharmacophores

Some antioxidants possess more than one type of the above structural features contributing to their activity. These include quinones which can be phenolic, but often use different mechanisms to generate their activity, such as isoprenoid-like chains in the common derivatives coenzyme Q10 (369) and MitoQ (370).

#### 3.5.1. Quinones

Quinones are a diverse group of chemicals containing a cyclohexadiene ring with two carbonyl groups. These groups can be *meta*- or *para*- to each other, and most quinones contain several substituents. Common natural quinones belong to four subcategories, anthraquinones (**351**–**364**), phenanthraquinones, naphthoquinones (**365**–**367**), and benzoquinones; naphthoquinones and anthraquinones being the most common. Other quinone derivatives, such as coenzyme Q10 (**369**) and MitoQ (**370**), have shown promise in their effectiveness of radical scavenging in mitochondrial systems.

##### Natural Quinone Derivatives

**Figure 25:**
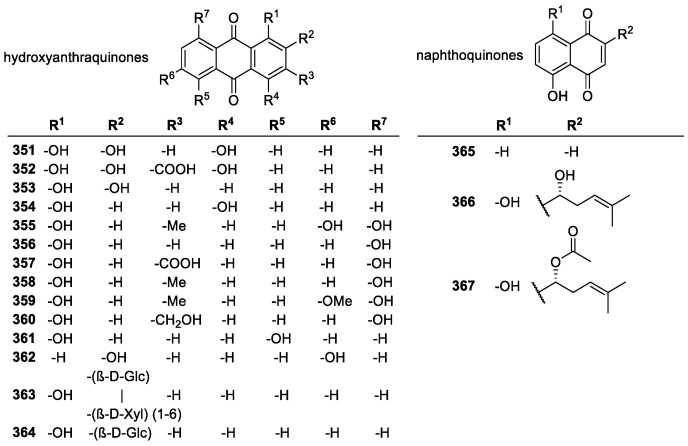
Structures of hydroxyanthraquinones and naphthoquinones.

Studies on quinones (Figure 25) have shown that they tend to display low or negligible antioxidant activity, especially when compared to other groups like flavonoids or cinnamic acids [210,211]. The general requirement for activity in anthraquinones is that they must contain an 1,2-dihydroxy unit, and that only one of the phenyl rings could be unsubstituted. Naphthoquinones were found to be nonactive as antioxidants in the same study [145]. In addition, the glycosylation of quinones reduced the activity of all quinones drastically [212]. Although, some studies have shown anthraquinones like emodin (**355**) to be active, if not potent [213], contradicting the above studies that suggested only the *ortho*-dihydroxyl substitution to be effective.

##### MitoQ and Coenzyme Q10

**Figure 26:**
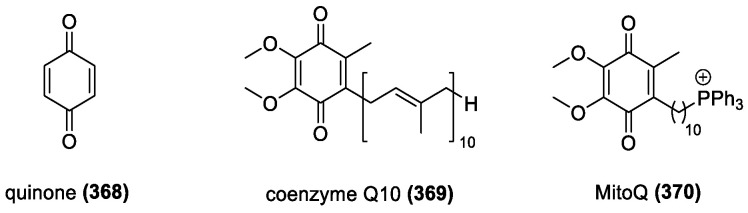
Structure of quinone, coenzyme Q10, and MitoQ.

Coenzyme Q (ubiquinone or CoQ) is an important constituent of plasma membranes in animals and other prokaryotes and is essential for electron transport [214]. It contains a 2,3-dimethoxy-5-methyl-1,4-benzoquinone skeleton and an isoprenyl chain in the 6-position [215]. Q10, one of the most studied ubiquinones, has a 10-isoprenyl unit chain. MitoQ is another ubiquinone with the same skeleton, although it has an alkane tail with a triphenyl phosphonium cation at the end of the chain (Figure 26).

Q10 has been shown to act as both a radical generator in its quinone form, and an antioxidant in its reduced alcohol state [216]. It has also been demonstrated to inhibit lipid peroxidation in vivo [217,218,219,220], likely due to its hydrophobic nature allowing it to stay within the membranes of mitochondria and other cellular and subcellular systems [221]. It also works with vitamin E as well, indicating a potentially synergistic effect [222]. Its mechanism of action is not fully known; suggestions range from its reduced form interacting with perferryl ions to generate hydrogen peroxide to be removed by other antioxidants, to direct interaction with lipid radicals, to more complex mechanisms involving oxidoreductase reduction and oxidization [223]. Q10 has been extensively studied for the treatment of various disorders, and it has been effective in reducing ROS from mitochondrial systems, in treating disorders linked to oxidative stress and damage [224,225,226], acting as a neuroprotective agent [227,228], and illustrating its important role in redox homeostasis and overall health of cellular systems.

MitoQ is a mitochondria-targeting ubiquinone driven by its triphenylphosphonium group. It is a potent antioxidant and effective in vivo [229]. The positive charge helps it to pass through the mitochondrial membrane [218,230]. In addition, MitoQ’s ubiquinone moiety stays in the lipid bilayer to be reduced to its ubiquinol form and protects against oxidative stress [231]. The length of the aliphatic chain influences hydrophobicity and efficiency of lipid binding, with smaller chains having negligible impacts, and larger ones being completely bound to the membranes. The chain has no effect on antioxidant activity as that is solely controlled by the ubiquinone ring [232]. In vivo it shows selective scavenging abilities and has been shown effective in the prevention of organ damage from hepatis, sepsis and other disorders [233,234,235], as well as improving vascular function and preventing damage to blood vessels [236,237]. In the case of all these quinones, it is the phenolic hydroxyl group that ensures the antioxidant activity, continuing the general trend of this group being one of the most important functional groups.

## 4. Effect of Physicochemical Properties on Antioxidant Activity

### 4.1. Electron Distribution—Aromaticity, Conjugation

One of the major factors seen in virtually every antioxidant is a high degree of conjugation. Nearly every compound discussed in this work is aromatic or at least has a high degree of conjugation. In general, highly conjugated systems are better able to stabilize radicals, since the overlapping π-bonds allow for electrons to be delocalized over the entire system instead of being localized on a single atom [137]. The more conjugated a system is, the better it will perform as an antioxidant. For example, carotenoids show high antioxidant activity despite their lack of phenolic OH or amino groups due to their chains allowing for high degree of delocalization. Extra electrons donated by EDG groups to already conjugated systems increase their ability to quench radicals by providing higher degrees of resonance, which in turn increases stability of the overall molecule and delocalizes the radical further.

### 4.2. Bond Dissociation Enthalpies and Substituent Effects

Since many antioxidants work by donating an H atom or an electron, the bond dissociation enthalpies (BDE) and ionization potential (IP) for the active XH bond can be evaluated to determine their potential activity. In phenolic antioxidants that work via the HAT mechanism, the donor capacity of the OH must be high, which in turn means that the BDE must be low [238]. Similarly, with ET mechanisms, the IP must be low [49]. BDE and IP values are important in determining the potential activity of antioxidants. The OH BDE values correlate very strongly with DPPH scavenging results, and even the second order reaction constant for DPPH activity, k_DPPH_ aligns well. Thus, BDE value calculations can be used to predict antioxidant activity, especially in new compounds [239].

The BDE of phenolic OH bonds are lower in the gas phase compared to phenolic bonds dissolved in polar aprotic solvents (87–90 kcal/mol vs. up to 95 kcal/mol), and depending on the substituents attached to the compound, the BDE and antioxidant activity can vary significantly [240]. When adding substituents to phenol, it was found that *ortho*- and *para*-positioning of EDG lowered BDE values and favored the HAT mechanism. This is due to the stabilization of the radical by adding to the electronic vacancy of the phenol or by adding to the conjugation of the compound, especially with *ortho*-positioned alkyls. The HAT mechanism is favored where there are adjacent groups that can undergo intramolecular hydrogen-bonding, as that tends to stabilize the radical and lower the BDE. The EWG at the same positions will destabilize the radical and increase the BDE, leading to a lower HAT capability and, therefore, antioxidant activity [240,241,242,243,244,245,246]. *Meta*-positioning of EDG has a minimal effect, however, and *meta*-EWG has a smaller negative impact [247]. Lower IP values favor SET, the extended delocalization of electrons and strong resonance and planarity of the molecules can be predicted [241].

Analyses of phenols revealed that the BDE of the OH in the unsubstituted phenol is around 86.7 ± 0.7 kcal/mol [248] and this is affected by the nature and position of substituents on the ring [249]. Potential intramolecular hydrogen bonding or steric interactions can affect the BDE, with hydrogen bonding between substituents potentially increasing the BDE. Substitution with alkyl groups caused a minor decrease of the BDE, down to 80.40 kcal/mol with a 2,4,6-trimethyl-substituted phenol. Methoxy groups (provided no intramolecular hydrogen-bonding is allowed to occur) will also lower the BDE. Amino groups had the largest decrease in the BDE, especially in a 2,6-pattern, as the amino groups can destabilize the phenol, but increase stability of the radical. Bicyclic systems were also tested, which in general had lower BDEs due to extended resonance. A general scheme of simple additivity rules can also be used to predict the decrease in the BDE, albeit with some exceptions based on general substitution rules for aromatic rings and hydrogen bonding [249]. Other studies have also found that electron-donating groups lowered the BDE of the hydroxyl groups and increased stabilization of the radical, while also increasing the ground state energy of the Ar-XH families, while meta and para withdrawing groups increase the BDE by making the radical less stable by lowering the ground-state energies [245,250,251]. This effect is shown for NH and SH BDE values, with the OH group having the largest increase [250].

In compounds more complicated than simple phenols, such as catechols, the BDE of the hydroxyl groups tend to be lower due to the substituent effect. In one report, the BDE of catechol is 72.82 kcal/mol, which is 10 kcal lower than that of phenol (82.83 kcal/mol). Even the removal of the hydrogen from the second hydroxyl group is comparatively easy with a BDE = 73.31 kcal/mol. The BDE changes for each substituent are slightly different for catechols vs. monophenols, but the overall trends are the same [252]. It has also been found that the calculated the BDE of several polyphenols with catechol units is often higher than the experimental value, indicating a strong cooperative effect. This is especially seen where adjacent hydroxyl groups can form a hydrogen bond with the radical and stabilize it [253]. This aligns with the general findings that catechols tend to be more potent antioxidants, since the hydrogens are significantly more easily abstractable than from monophenols. In fact, this effect can be extended to flavonoids, the most active of which contain a catechol moiety and resonance between the individual rings in the system. For example, the conjugation of luteolin (**156**) is higher than catechin (**196** *trans* isomer), but catechin is found to have a lower OH BDE in ring B, which corresponds to its higher antioxidant activity. This would appear to be an anomaly, but in fact corresponds to the rest of the data that EWG groups, like the pyrone structure of luteolin, raise the BDE, and therefore make hydrogen abstraction more difficult [252]. In polyphenolic antioxidants, many of the compounds with the lowest OH BDEs are those previously mentioned above to have high scavenging abilities. The effective flavonoids and many ubiquinols have extremely low BDEs compared to alpha-tocopherol, and they are all known to be effective scavengers. Their structures are well made for delocalization, and especially compounds like quercetin and epicatechin have the ideal catechol moiety and resonance ability to be potent antioxidants due to their multiple possible active sites [254].

Resveratrol (**65**) has a low BDE, despite the lack of intramolecular hydrogen bonds. Due to its planar structure and low IP it might be expected to act under the SET mechanism. However, some of its stilbene-based analogs have higher IP values than alpha-tocopherol, which reacts mainly by the HAT [241,247]. In some cinnamic acids, modifying substituents can lower the BDE and IP of the phenolic hydrogen. In ferulic acid analogs the aldehyde vs. alcohol forms had noticeably different BDE values, indicating that the effects of the EWG and the EDG extend throughout the entire molecule [255].

Nitrogen containing antioxidants typically have low NH BDE values, even when compared to alpha-tocopherol, indicating strong HAT-based activity [256]. Substituent effects of the EDG and the EWG on the BDEs of NH groups in anilines are known to follow a similar pattern to that of phenols, albeit the effects are not as strong. The effect of ED substituents on phenolic radical stabilization is much greater than that of the NH^∙^ radical [257]. The reason for the higher activity aided by the EDG can be determined through the analysis of the pyramidalization of aniline derivatives. When more electrons are placed into the system, the effect is increased, causing the NH bond to overlap more with the aromatic ring system, weakening the bond, thus allowing for the hydrogen to be abstracted more easily, making scavenging more pronounced [258].

### 4.3. HOMO and LUMO Calculations

The HOMO—LUMO gap energy is often used to help predict antioxidant activity in several compounds. While some studies show that it is not always relevant [163], there is evidence to its usefulness in predicting scavenging ability. The Highest Occupied Molecular Orbital (HOMO) is essentially an electron donor to the Lowest Unoccupied Molecular Orbital (LUMO), with their energies being related to ionization potential and electron affinity, respectively [259]. HOMO energies are known to be useful for determining the electron donating ability of a compound, with higher values being more favorable. A greater HOMO—LUMO gap has been positively correlated with overall ABTS scavenging ability in phenols and anilines, which combined with larger HOMO values, indicates a higher likelihood of the SET mechanism for activity. Similar trends are seen with aniline-based antioxidants [163]. By determining the HOMO—LUMO gap of potential antioxidants, it could be possible to predict their activity and see if any trends can be found with different substituent groups.

## 5. Strategic Structural Modifications for Improving Antioxidant Action

### 5.1. Limitations of Therapeutic Efficiency—The Need for Modifications

Several antioxidants discussed in this paper are of therapeutic interest. For example, glutathione, melatonin, bilirubin, ubiquinone, vitamin C, and vitamin E are all known to be involved in human cellular systems. Flavonoids and other polyphenols have been considered for therapeutic use due to their high antioxidant potency despite their limitations, and as such several modifications should be made to them before they can be considered effective in vivo. These modifications can include improving the activity of the pharmacophore, making them more lipophilic, or even modifying them for mitochondrial targeting. Any potential modification that sees a large increase in activity, especially in vivo, has the potential to be game changing, and as such the drive to create new antioxidants via structural modifications, or to create novel molecules, is extremely high.

### 5.2. Improving Pharmacodynamic Properties/Potency

As previously discussed, the addition of an EDG tends to have a generally positive effect on antioxidant activity. One of the most important factors in predicting antioxidant activity is the HOMO—LUMO gap. Strategically placed EDG or EWG groups can change the total antioxidant activity, affecting the BDE, IP, and potentially the HOMO—LUMO gaps. Additional aromatic and conjugated groups can have a potentially positive effect as well, allowing for more resonance stabilization, potentially more active groups, or even combined mechanisms.

A potential modification that can greatly affect potency is the synthesis of imine analogues of stilbenes. This modification replaces a carbon in the bridge with a nitrogen and has been shown to greatly increase activity of resveratrol analogues [105,106]. Should this be done on other molecules, the potential of developing successful therapies based on these novel antioxidants could increase significantly. Addition of sugar groups to some polyphenols has had some success in increasing activity as well, so attaching similar groups, or at least other larger groups, to different antioxidants could have beneficial effects as well.

### 5.3. Improving Pharmacokinetic Properties

#### Log *p* and Lipophilicity

The partition coefficient (*p*) and its log *p* values are used to ascertain a compound’s favorability for organic or aqueous media, with higher values being more favorable to organic media. In terms of antioxidant activity, this could be useful as several known antioxidants work by preventing lipid peroxidation (carotenoids, vitamin E, MitoQ) and are highly lipophilic and have long alkyl or conjugated chains. Many other antioxidants, however, are highly hydrophilic, and do not dissolve well in organic media. As ROS damage often affects lipid-rich cellular membranes, it is desirable to have more antioxidants work under these conditions. Therefore, improving log *p* values and lipophilicity of antioxidants is one potential method of improving overall efficacy of ROS scavenging for normally hydrophilic compounds such as polyphenols.

The process of improving lipophilicity must be done carefully to maintain antioxidant activity. It includes grafting alkyl chains onto the antioxidant molecule in such a way that they do not interfere with the active pharmacophores [260]. The typical spot for esterification is on carboxylic acid groups, since those tend to not be directly involved in antioxidant activity [261]. There is a known paradoxical relationship between chain length of alkyl esters and antioxidant effectiveness, with polar antioxidants being more active in oil-water emulsions. This “polar paradox” has been studied, and it has been found that it is more a matter of chain length, with the relationship between chain length and activity being nonlinear, and indeed parabolic. Several studies have shown that octyl chains are often the maximum length before antioxidant activity starts to decrease. As such, medium chains are thought to be optimal for creating novel phenolipids [262,263,264,265].

Several studies on lipophilization of polyphenolic antioxidants have been conducted. The general trend was that addition of properly positioned lipophilic chains do not decrease their overall scavenging abilities but rather increase activity in non-polar systems. For example, ester derivatives of epigallocatechin gallate, already a strong antioxidant, showed high incorporation and strong protecting abilities in lipid-rich systems. Some studies show little loss of overall potency when compared to the parent molecule as well, indicating that proper application of lipophilic chains will not affect the active pharmacophores [266,267,268,269,270]. Others, however, show less overall scavenging in compounds with improved lipophilicity due to potential loss of active hydroxyl groups, but overall efficacy in preventing lipid peroxidation was much higher compared to the parent phenols [271]. Similar results have been shown for dihydrocaffeic acid [272], hydroxytyrosol [264], 4′-acetylated resveratrol [273], rutin/fatty acid derivatives (which also show that longer chains prevent onset of peroxidation even with lowered scavenging) [271], quercetin [274], gallic acid [275], and anthocyanins [276,277,278].

In terms of reaction kinetics, it was found that within 2 h, most chain lengths of 0 (parent phenol) to 10 carbons were found to have about the same level of efficacy, with longer chains performing significantly worse and taking longer to reduce ROS levels. After 24 h, however, the chain lengths of 8–10 carbons were found to be the most effective overall, meaning that in addition to being fast acting, they have longer viability [269]. Log *p* values have also been measured and compared to activity, and although the longer chains typically are more lipophilic, the cut-off for activity seen with the rutin derivatives implies that a log *p* of around 0 is correlated to the highest antioxidant activity. The compounds with values around 0 tended to be chains of 8, 10, or 12 carbons in lengths, with 10 being the closest to 0, which corresponds to the cut-off value from the polar paradox [271].

Based on the above, improving lipophilic character should be considered as a potential tool in designing novel antioxidants for therapeutic use. With the higher lipophilicity on par with vitamin E, these compounds could be used for the treatment of various disorders far more effectively than the current treatment options.

### 5.4. Targeted Delivery

#### Mitochondrial Delivery

One important aspect of antioxidant therapy is the ability to target a specific region in a biological system. While lipophilicity can certainly help with targeting lipids in cells, it lacks specificity. Most antioxidants do not target just one kind of cell or lipid system. If antioxidants could only work, or prefer to work, in specifically targeted areas, it could help prevent cellular damage on a much more precise level, increasing efficacy of new therapies. For example, mitochondrial disorders often have limited therapies, and the use of antioxidants to prevent oxidative stress are being evaluated as therapeutic options. Some disorders such as Parkinson’s and Alzheimer’s have been linked to mitochondrial damage, and the need to mitigate or even prevent such damage is high [279,280].

MitoQ, melatonin, and Q10 target mitochondria, and thus are of particular interest in treating mitochondrial disorders and ROS damage. In MitoQ, the main reason for this is the TPP end of chain. The TPP cation combined with the long lipophilic chain make it easy for compounds like these to specifically target certain areas of cells and prevent oxidative damage in mitochondria that have a highly negative membrane potential. In addition to the increase in lipophilicity from adding alkyl chains, TPP could be added to other known antioxidants to allow them to target mitochondria. This could open new avenues for treating specific disorders and prevent oxidative damage in the mitochondria. By testing compounds with an esterified TPP group, it can be determined what the effect on the original scavenging ability is, and if they do in fact selectively target mitochondria. The modification of existing molecules, such as resveratrol, for mitochondrial targeting has been studied. The esterification of 4′-OH group of resveratrol with a TPP containing group, the analogues were able to selectively target mitochondria, although they tended to act as pro-oxidants at this stage with high cytotoxicity [281,282], which makes them useful for targeting cancer cells. Similar effects were found with TPP modified curcumins [283] and quercetin [284,285].

The purpose of these changes was to enact cytotoxic effects, but as seen with the synthetic MitoQ, it could be possible to find a modification with TPP that enhances antioxidant activity. Such molecules could be useful against mitochondrial disorders, which currently have minimal treatment options available.

## 6. Summary and Conclusions

The use of antioxidants for therapeutic purposes has been of great interest in recent years, although practical applications are hampered by the lack of in vivo mechanisms. While there are known antioxidants that can target human cells and provide protection against various ROS, modifications of known antioxidants are necessary for development of new therapeutic approaches. In this work we summarized the main structural features that are responsible for antioxidant activity and reviewed representative compounds in each group. During this survey we also highlighted structural indicators in the compounds that lead to higher or lower antioxidant activity. While the high degrees of conjugation and aromaticity as key components of most antioxidants are well-known, it appears that the number, the individual position, and state of derivatization of the XH groups significantly affect the antioxidant activity. In addition, we also highlighted the negative and positive effects of electron-donating or electron-withdrawing substituents present in the compounds. The importance of substituent effects is also highly essential to activity, with affecting resonance stabilization. These structural features have been correlated with experimental antioxidant activity in structure—activity relationship descriptions for each compound group. We have also reviewed the effect of physicochemical variables, such as HOMO—LUMO energy gaps, bond dissociation energy, and ionization potential on the antioxidant activity. All of these have been correlated with scavenging ability to better understand these relationships. In the last part of the work methods to improve antioxidant activity were surveyed, which can lead to the development of improved therapeutic antioxidant agents.

## Data Availability

No new data were created.
